# Exclusion of context knowledge in the development of prehospital guidelines: results produced by realistic evaluation

**DOI:** 10.1186/1757-7241-21-46

**Published:** 2013-06-22

**Authors:** Magnus Andersson Hagiwara, Bjorn-Ove Suserud, Anders Jonsson, Maria Henricson

**Affiliations:** 1 University of Borås, School of Health Sciences, 501 90, Borås, Sweden; 2 Jönköping University, School of Health Sciences 551 11, Jönköping, Sweden

## Abstract

**Background:**

Prehospital work is accomplished using guidelines and protocols, but there is evidence suggesting that compliance with guidelines is sometimes low in the prehospital setting. The reason for the poor compliance is not known. The objective of this study was to describe how guidelines and protocols are used in the prehospital context.

**Methods:**

This was a single-case study with realistic evaluation as a methodological framework. The study took place in an ambulance organization in Sweden. The data collection was divided into four phases, where phase one consisted of a literature screening and selection of a theoretical framework. In phase two, semi-structured interviews with the ambulance organization's stakeholders, responsible for the development and implementation of guidelines, were performed. The third phase, observations, comprised 30 participants from both a rural and an urban ambulance station. In the last phase, two focus group interviews were performed. A template analysis style of documents, interviews and observation protocols was used.

**Results:**

The development of guidelines took place using an informal consensus approach, where no party from the end users was represented. The development process resulted in guidelines with an insufficiently adapted format for the prehospital context. At local level, there was a conscious implementation strategy with lectures and manikin simulation. The physical format of the guidelines was the main obstacle to explicit use. Due to the format, the ambulance personnel feel they have to learn the content of the guidelines by heart. Explicit use of the guidelines in the assessment of patients was uncommon. Many ambulance personnel developed homemade guidelines in both electronic and paper format. The ambulance personnel in the study generally took a positive view of working with guidelines and protocols and they regarded them as indispensable in prehospital care, but an improved format was requested by both representatives of the organization and the ambulance personnel.

**Conclusions:**

The personnel take a positive view of the use of guidelines and protocols in prehospital work. The main obstacle to the use of guidelines and protocols in this organization is the format, due to the exclusion of context knowledge in the development process.

## Introduction

One major threats to patient safety in the prehospital setting is errors in the decision-making and clinical judgment [1-3]. Possible reasons for errors in decision-making include the significant change in prehospital practice in recent years and the fact that more advanced care is delivered. Examples of more complex interventions include the new cardiopulmonary resuscitation (CPR) process for cardiac arrest, ST-elevation myocardial infarction identification and transport bypass protocols, early stroke identification and transport bypass protocols and therapeutic interventions in trauma [4].To support the prehospital providers in their decision-making process, protocol-based care is a necessity, but there are signs that prehospital protocols are not always suited to the prehospital setting [1]. There are a number of studies which have shown poor compliance with prehospital guidelines and protocols [5-10], but there are few studies that have investigated the reasons for the poor guideline compliance in the prehospital setting. Reasons for poor compliance could be lack of communication, standardization, education and local implementation strategies and that guidelines and protocols are difficult to understand [2,11]. In a previous simulation study, prehospital guidelines using a Computerized Decision Support System (CDSS) were compared with a standard paper-based version of the guidelines and showed significantly greater compliance with the guidelines when using the CDSS [12], but the study was not able to determine why there was a difference in compliance. At this point, there are few studies that have explicitly investigated the use of guidelines and protocols in the prehospital context. The aim of this study was to describe how guidelines and protocols are used in a prehospital context, who uses them, why they are or are not used and under which circumstances they are used.

## Methods

### Study setting

In order to collect extensive data, the case was sampled from an ambulance service with urban and rural stations. The stations were involved in a simulation study where guidelines built into a CDSS were compared with the use of guidelines in the standard paper format [12]. Two types of personnel are employed in the organization, Emergency Medical Technicians (EMT), who have a shorter education and do not perform all the types of treatments in the organization, and Ambulance Nurses (AN), who are registered nurses with different kinds of specialist education. For demographic data see Table 1. Several different types of guideline and protocol (Table 2) were used in the study [13,14].

**Table 1:** Characteristics of interview, observation and focus group study subjects

**Phase**	**Number of participants**	**Age**	**Gender**	**Education**	**Years in ambulance service**
**Phase 2 Semi- structured interviews**	n = 3		Male n = 2	Doctor n = 1	
			Female n = 1	Nurse n = 2	
**Phase 3 Observation rural station**	n = 11	Mean = 42	Male n = 7	Emergency Medical Technicians n = 1	Mean = 14
		Range = 32-58	Female n = 4	Ambulance nurse n = 10	Range = 1-34
**Phase 3 Observation urban station**	n = 16	Mean = 42	Male n = 13	Emergency Medical Technicians n = 1	Mean = 12
		Range = 32-58	Female n = 3	Ambulance nurse n = 15	Range = 2-32
**Phase 4 Focus group rural station**	n = 8	Mean = 42	Male n = 6	Emergency Medical Technicians n =0	Mean = 12
		Range = 33-50	Female n = 2	Ambulance nurse n = 8	Range = 5-28
**Phase 4 Focus group urban station**	n = 5	Mean = 44	Male n = 3	Emergency Medical Technicians n = 0	Mean = 14
		Range = 36-54	Female n = 2	Ambulance nurse n = 5	Range = 10-25

**Table 2:** Guidelines and protocols used in the organization

**Name of the guideline/protocol**	**The main guideline**	**The pocket guideline**	**The triage protocol “METTS”**	**Pathway protocols**
**Format**	194 A4 pages in a file. The file did not contain any register.	File in A5 format. Contains no register.	Two formats: the “old” METTS is in an A5 format file and the new METTS in an A4 file. Connected to a patient record file for use as support when handling over the patient.	A4 papers in a file with 5 different pathway protocols.
**Content**	Description of assessment of the medical and trauma patient. Description of different symptoms and conditions and directions for treatment for both adults and children. Description of drugs. Description of some local procedures and routines.	Tables of drug doses, normal values and a few algorithms, such as cardiopulmonary resuscitation.	A mixture of symptoms and diagnoses. The symptoms or diagnoses have their own page with an algorithm which describes a triage grade based on different symptoms. The patient is given a triage color based on symptoms and vital parameters.	Some pathway protocols take the form of checklists with boxes to tick, while others are plain, descriptive text.
**Location**	Between the seats in the front of the ambulance and one copy in the back of the ambulance.	In an ambulance staff member’s leg pocket.	In the back of the ambulance.	In the back of the ambulance.
**Development process**	Informal consensus	Informal consensus	Unknown	Unknown

### Study design

The methodological framework in the study is realistic evaluation [15]. In realistic evaluation, the context is an important factor when it comes to understanding why an intervention works, for whom, why and under which circumstances. The theory of realistic evaluation is that underlying mechanisms are always embedded in particular contexts and it is necessary to understand these mechanisms and the effect context has on outcomes. The connection is called Context, Mechanisms, Outcomes (CMO) configuration. Realistic evaluation is a theory-driven approach where theory guides the researcher through the evaluation process (Table 3). The theory conducts the conjectured CMO configuration and, through a process of accumulation through different phases in the study, the goal is to construct the refined CMO configuration which is the final result of the study. To be able to understand the context, multiple sources of data were collected according to the case study design [16].

**Table 3:** Evaluation steps in realistic evaluation


1.	Literature screening and creation of a theory
2.	Construction of a conjectured Context, Mechanism, Outcome (CMO) configuration
3.	Test of the conjectured CMO configuration by data collection
4.	Presentation of a refined CMO configuration (middle range theory)

### Data collection

The case study was divided into four separate phases. As a theoretical framework, the Promoting Action on Research Implementation in Health Services (PARIHS) framework [17] and the study of “protocol-based care” in the United Kingdom, performed by Rycroft-Malone et al. [18], was used.

Phase 1: A literature screening was performed and the result of the screening was used to produce the first conjectured CMO configuration and was also the basis of the interview guide, observation protocol and code template.

Phase 2: In phase two, three semi-structured interviews with stakeholders involved in the development, implementation and maintenance of the organization’s guidelines and protocols was performed. The informants were collected using a convenience sampling process. The semi-structured interviews were conducted using an interview guide constructed from the theoretical framework from phase one and they lasted between 40 and 60 minutes. The interviews were conducted by one of the authors (MAH). In addition to the interviews, sampling of relevant documents, such as guidelines and protocols, was done. A new conjectured CMO configuration was produced after this phase.

Phase 3: In the third phase, 30 participant observations were made, 15 at an urban ambulance station and 15 at a rural ambulance station. The sampling could be described as random though the researchers were participants in the ambulance missions that took place. Two separate researchers both of whom familiar with the prehospital setting conducted the observation and were guided by an observation protocol based on the theoretical framework. In relation to the observations, short semi-structured interviews were carried out with the aim of clarifying some questions during the observations. The interviews lasted between 5 and 10 minutes. The conjectured CMO configuration was rebuilt after phase 3.

Phase 4: In the last phase, two focus group interviews took place. The first focus group interview was held at the rural station and the participants were collected using a convenience sampling process with the aim of obtaining a distribution in the participants’ education and experience. A rebuilt interview guide was used and the interview was conducted by three of the authors (MAH, AJ, MH). The interview lasted about 70 minutes and was used to produce a new conjectured CMO configuration. The second focus group interview can be described as an “analytic” focus group [19]. It was conducted at the urban station and, with the help of the participants, it aimed to refine preliminary data from the earlier phases and, at the same time, function as a member check [20]. The session started with a presentation of the preliminary data on a screen and the participants were asked to comment on the data that was presented. The interview lasted about 60 minutes and was conducted by two of the authors (MAH, MH).

### Analyses

To construct and refine the CMO configuration, a template analysis style [20] was used in the analysis of interviews, guidelines, protocols and observation protocols. A code manual based on the theoretical framework of the study was developed [17,18]. The manual was pilot-tested on two independent researchers who were not involved in the study and it was refined before it was used in the study. Complete interviews and observation protocols were regarded as units of analysis. The units were read in their entirety several times and segments of texts which were regarded as answering the research question were highlighted. The highlighted segments were then attached to a predefined code in the code manual. If there were no suitable codes for a highlighted segment, a new code was developed. The coding was performed by one of the authors (MAH). When the text of an analysis unit was coded, a chunking process began. Texts which were similarly coded were put together in chunks. The chunks were analyzed with an immersion/crystallization style [20] where the chunks were read several times and categories were identified. This process was conducted by two of the authors (MAH, MH). A summary of the categories was displayed in a matrix [21]. The matrices in a given phase were summarized in a phase matrix. Using an accumulation process, the matrix was rebuilt in every phase. The process was iterative, where comparisons with data in the previous phase are compared with data in the next phase to construct the refined CMO configuration. In the accumulation process, four authors (MAH, MH, BOS and AJ) were involved and the refined CMO configuration was made by consensus between the four authors.

### Validity

The internal validity was maintained by pattern matching against the theoretical framework and theory triangulation. Construct validity was preserved by a process of data triangulation (archival data; interview data; participatory observations) [16] and member checking [20].

### Ethics

Informed consent was obtained from all the participants in the study. The study was approved by the Regional Ethics Committee, Gothenburg, Sweden (Dnr: 1133–11).

## Results

The main finding in the present study was that, during the development of the guidelines, no professionals with context knowledge were involved in the process. One result of this shortcoming is a poorly adapted format which leads to the implicit use of the guidelines. The result as a whole is presented in categories and sub-categories. A summary is presented in Tables 4 and 5.

**Table 4:** What works for whom, how and under which circumstances


**What works**	– Guidelines with a degree of force
	– Guidelines which are connected to patient notes
	– Guidelines in a format adjusted for use outside the ambulance
	– Guidelines in a format where it is easy to look up information
	– Guidelines with a degree of flexibility
	– Systems which collect all the information in one place
	– Implementation strategy based on education, simulation and interactive activities
	– Development of guidelines involving people with context knowledge
**For whom**	– Both experienced and inexperienced ambulance personnel
	– The team, who can work on a common strategy
	– The patients, who are treated more equally
**How**	– The guidelines are mostly used implicitly because of the format
	– Protocols with some form of checklist or algorithm are used more explicitly
**Under which circumstances**	– On route to the patient as preparation
	– Outside the ambulance to check medical doses
	– In the ambulance during transport to triage the patient
	– When the patient is part of a pathway

**Table 5:** Refined CMO configurations

**Context**	**Mechanism**	**Outcome**
Context 1: The prehospital work is accomplished a long distance from medical support; the personnel handle many different symptoms and conditions in changing environments and they have different levels of education and experience.	Mechanism1: The guidelines are developed in a process in which people with in-depth context knowledge are excluded.	Outcome 1: Guidelines with a format poorly adjusted to the context.
		Outcome 2: Guidelines with limited explicit use.
		Outcome 3: Development of homemade guidelines.
		Outcome 4: Lack of organizational control.
		Outcome 5. Explicit use of guidelines in file format creates a sense of unprofessionalism.
	Mechanism 2: Structured implementation strategy of the guidelines at local level.	Outcome 1: The personnel are well informed of the guidelines and their content.
		Outcome 2: The personnel take a positive view of the guidelines.
		Outcome 3: Both inexperienced and experienced personnel use them.
		Outcome 4: Improved team function.
	Mechanism 3. Difficulty developing guidelines which cover every possible situation.	Outcome 1: Deliberate deviation from guidelines.
		Outcome 2: Ethical conflicts.
	Mechanism 4: The ambulance mission is divided into 5 separate phases.	Outcome 1: Different need for support in the different phases.
		Outcome 2: Request for a system which covers all phases.

The table show the mechanisms embedded in the context and theirs effects on outcome.

### Development

This category describes how guidelines are developed.

#### Exclusion of context knowledge

The development of the guidelines could be described as an informal consensus approach. The process comprises three steps. The first step is at national level where the medical controllers from different regions of Sweden meet and discuss the content of the national prehospital guidelines. The medical controller from each region then takes the national guidelines home and makes some adjustments based on regional differences. In the last step, the regional guidelines are delivered at local level where the local organization makes the final adjustments based on the local context. There is very little adjustment from national to local level. During this process, the actual users of the guidelines (EMT and AN) have very little or no impact on the development of the prehospital guidelines, *“Yes, they can give me or the local medical controller their points of view, but in the end there is not much opportunity to influence things, they have already been decided in the region”* (regional medical controller). Instead, the development is done by people with limited knowledge (hospital-based doctors) of the prehospital context.

### Implementation

This category describes how guidelines are implemented in the local organization.

#### Well-thought-out local implementation

At local level, there is a conscious implementation strategy. There are continuous education opportunities with lectures and manikin simulation based on the content of the guidelines and the ambulance personnel are obliged to perform an IT-based test to obtain certification based on the local guidelines. There is also a facilitator in the organization that has a clear-cut role of implementing guidelines and protocols.

### The ambulance personnel’s perception of working with guidelines

This category describes the ambulance personnel’s view of working with guidelines in the prehospital setting.

#### An absolute necessity

In general, the ambulance nurses and EMTs take a very positive view of the use of guidelines and protocols. They regard them as an absolute necessity and their existence is not questioned. This perception is the same for both the experienced ambulance nurses and the more inexperienced ones. *“I like them; they are a good support in some situations. If we are in a hurry and the patient is extremely ill, I don’t use them, it takes too much time, but I usually read them when things are more stable. I think you can learn a lot from them”* (AN, 13 years’ experience of the prehospital field). The ambulance personnel regard the guidelines as a way of being able to work with advanced health care a long distance from medical support.

#### A part of quality improvement

The ambulance personnel are generally concerned about quality and quality improvement issues and regard the guidelines and protocols as a way of making the care more uniform and giving the ambulance team a common structure to work with. *“They (the guidelines) give us a common language when we communicate with one another” (AN, rural station).* The users of the guidelines and protocols want to be more involved in the development process of the guidelines. They mostly comprise issues such as working processes and formats they would like to influence. *“We detect the shortcomings in the guidelines, we can’t decide about the kind of medication to use, but I think it’s important for us to be able to influence the format and way of working”* (AN, rural station).

#### Lack of evidence

Another area of concern is the lack of evidence in the guidelines. In spite of the fact that the ambulance nurses think that the guidelines have a positive effect on the outcome, they are seldom regarded as evidence based. The ambulance nurses are sometimes frustrated by the fact that the patient can be given a different assessment and treatment at the hospital, which gives the feeling of a low level of evidence. “*We have different guidelines compared with the hospital. If, for example, we handle a patient with a suspected stroke, they sometimes make a different judgment at the hospital based on their guidelines”* (AN, urban station)*.* One exception is the protocols used in cardiac arrest which are upgraded at regular intervals based on new evidence and this creates a sense of quality. Another element of the good design of the cardiac arrest protocol is the fact that end users are involved in the development process.

#### Deviation from guidelines

The prehospital context forces the personnel to work with a high level of flexibility and creativity. On many occasions, they have to handle situations which are not actually part of the ambulance service’s normal mission. *“We can end up in situations where we are supposed to solve everything, we have to deal with things we should not be dealing with”* (AN, urban station). Following guidelines too literally is thought to be a threat to creative thinking. The view of the guidelines is that it is not possible to follow them in every situation and it is not possible to create a guideline for every situation in the prehospital setting. Deviations from guidelines are most common in situations where there are ethical issues, in connection with cardiopulmonary resuscitation, for example.

### Use of guidelines during the ambulance mission

This category describes the use of guidelines and protocols during ambulance missions. The ambulance mission is clearly divided into five different phases: 1. Receiving the call and driving to the patient, 2. First contact and assessment, 3. Transporting the patient to the ambulance, 4. Transport to hospital and 5. Handing over the patient to the hospital and patient record documentation. There is a need for different kinds of support in the different phases. The guidelines and protocols physical format is the aspect which has the greatest influence on the use.

#### Explicit use

The only guideline format which can be used explicitly in rare cases in the first patient assessment is the guideline in pocket format and in this case it is only used to check and repeat some medical doses. One exception is when the patient could be the subject of a clinical pathway. A pathway is an example of a case in which many different systems have to be used and the team has to work together in order to sort everything out. *“There are many papers to handle. Both the patient record and the pathway protocol have to be filled in. The parameters are written down in different places. There is a lot to do with all the assessments, questions, documentation and phone calls.”* (observation protocol, urban station). During the transport to hospital, one protocol is dominant. This is the triage protocol, “METTS”. The reason for the explicit use of the protocol is the fact that the ambulance personnel are obliged to give the patient a priority and the protocol is also linked to a paper-based patient record. The personnel do not regard the protocol as a prehospital protocol; it is more of a service for the emergency department. At the same time, the ambulance personnel can obtain some useful support from the protocol, mainly when, according to the protocol, the patient is given a higher priority than the ambulance personnel had initially supposed*. “The patient is given red according to METTS and this makes you to think again, but I can’t say that it actually influences the assessment”* (AN, urban station). Another reason for the explicit use of the triage protocol is the format. *“That’s one reason why I use it; it’s small and easy to look up. You don’t usually need all that information in the guidelines, just algorithms that can help to jog your memory.”* (AN, rural station).

#### Implicit use

The main guidelines are very seldom used explicitly and never during the patient assessment. The format makes it difficult. The guidelines are too large to carry around and the ambulance personnel are not comfortable using them in direct contact with the patient. *”You have to be familiar with the content of the guidelines in your head. It creates a feeling of unprofessionalism if you read that large file in front of the patient”* (AN, urban station). Sometimes the team can discuss the content of the guidelines on the way to the patient as a preparation. This is more common at the rural station. The main guidelines are generally used during education and as a textbook after a complicated mission.

### Critics of the guidelines request new formats

This category describes a need for a system that can guarantee the quality of patient assessment and the organization’s request for quality control.

#### Quality and control

The varying needs for support during the different phases has resulted in a request for a system that is able to collect all the different guidelines and protocols in a single system. It should be possible to use the system directly in the first contact and at the same time start the documentation. *“It would be beneficial if we could begin the patient record at the same moment we start the assessment and get some support at the same time”* (AN, rural station). The ambulance personnel in the study did not think that these requests could be realized in paper format. From the organization’s perspective, there is a need to have control of compliance with the guidelines. There is a lack of feedback from the present system. Deviations from guidelines probably exist, but they are never reported and the quality of the prehospital patient records is therefore unsatisfactory. One way to improve compliance with the guidelines is to have a greater degree of control. Electronically based guidelines connected to patient records can give this control *“For my part, I would be comfortable if there were a system where it was not possible to do things differently or make deviations, because the system would notice this”* (leader of the ambulance organization).

#### Development of homemade guidelines

One sign of format problems is the fact that many ambulance nurses make their own variety of guidelines, in paper format but most commonly in electronic format. Homemade guidelines in paper format can be files with a mixture of medical tables, algorithms and useful information. It is common for the personnel to have the guidelines in their own smartphone, *“I always have it with me (private smartphone) and I can just press a button and access the entire guidelines.”* (AN, urban station). These guidelines are available during every phase of the mission. The problem is that the guidelines do not have a format suited to smartphones and this means that they can be difficult to operate. The smartphone is also used to search for information on the internet.

## Discussion

The main obstacle to guideline use in this organization was the format of the guidelines, which was not suitable for the prehospital environment and the specific content of an ambulance mission. The fact that no one with context knowledge was involved in the development process is probably the reason for the insufficiently adapted guidelines. Earlier studies support this finding of the need for local ownership [22] and, in the Appraisal of Guidelines for Research & Evaluation (AGREE) Instrument, one item is “*The guideline development group includes individuals from all relevant professional groups*” [23]. Context knowledge is probably even more important in the prehospital context where the care is given in an unstable environment. In addition to involving people with context knowledge, the developers must have knowledge of the way ambulance personnel cognitively and practically make decisions in a natural setting [24]. Decision-makers in a natural setting follow a common cognitive process based on a combination of experience and mental simulation [25] and guidelines need to be adapted to the decision-making process in a dynamic environment. Kahol et al. [26] argue that all deviations from guidelines in a natural environment are not always errors. Deviations can sometimes be defined as innovations and can be used as a way to improve the guidelines.

Except cognitive errors and errors in clinical judgment [1,27] there is also a substantial risk of information being lost in the handover phase [28]. One method to reduce these patient safety issues is clinical guidelines in a format permitting explicit use in the patient assessment built into a system connected to a patient record. Both the ambulance personnel and the organization management in the present study request guidelines in another format. The management has a need for more control and the personnel request a system to support the assessment process. The ambulance service management’s perception that errors in the prehospital setting are underreported is supported by previous research [29]. Electronic medical records (EMR) with decision support can be a format suitable to match this request. Well-designed EMR can enable the ambulance personnel to collect information in a more systematic way and reduce some of the cognitive workload and reduce information lost [30].

The local implementation of the guidelines was based on education which involved simulation training. Previous studies of successful implementation reveal that interactive education can result in positive changes in practice [31]. Successful local implementation can be one reason for the ambulance personnel’s positive perception of using guidelines.

Lack of evidence in the guidelines has been an obstacle to guideline use, especially among physicians [18,32]. The lack of evidence in the prehospital guidelines was a matter of concern among the ambulance personnel, but the level of evidence did not have an effect of the use of guidelines. The guidelines are what they are and they have to use them. The level of evidence in prehospital guidelines is a problem worldwide. Most of the evidence in prehospital guidelines comes from research in hospital; approximately 4% of the evidence in prehospital guidelines originates from high-quality prehospital studies [33]. The present study was observed ANs and EMTs usage of guidelines. How the results could be in a physician-based service is difficult to predict. Earlier studies on guideline use in other settings shows that physicians are more skeptical to guidelines compared to nurses [32]. In a study of compliance to prehospital guidelines of hypertonic saline use the compliance rate among the prehospital physicians was low. Mostly it depended on that the physicians consider the evidence insufficient [34]. Physicians seem to be more sensitive to the level of evidence in guidelines in comparison to other groups.

### Limitations and further research

The present study is a single-case study. A single-case study is regarded as a weaker method compared with a multiple-case study where it is possible to compare the results between the cases [16], but the single-case study can produce better description of the context [35]. In order to collect more extensive data, the present study data were collected at two ambulance stations, but both share the same guidelines, protocols and organization. It might be possible to present a wider picture, if the results could be compared with different ambulance organizations with different guidelines and protocols. The chosen methods with qualitative data make the result difficulty to generalize. But generalization is not the goal of qualitative research; instead the goal is exploration of social phenomena [36] and together with a deep context description the result can be transferred to other settings.

## Conclusion

The ambulance personnel take a positive view of working with guidelines, but the format of present guidelines and protocols is the main obstacle to their use. A system used in the direct assessment of the patient, where the personnel simultaneously can begin the patient record at the same moment, might be a way of increasing compliance with guidelines. It is important that people with context knowledge are involved in the process of developing prehospital guidelines.

## Competing interest

The authors declare that they have no competing interest.

## Authors’ contributions

MAH was designing the study, participated in interviews and observations, participated in the analyses of data and drafted the manuscript. BOS was participated in the analyses of data and drafted the manuscript. AJ was participated in interviews, participated in the analyses of data and drafted the manuscript. MH was participated in interviews, participated in the analyses of data and drafted the manuscript. All authors read and approved the final manuscript.
